# Impact of baseline risk of death or hospitalization on effectiveness of revascularization in patients with ischaemic left ventricular dysfunction—a prespecified analysis of REVIVED-BCIS2

**DOI:** 10.1093/ehjqcco/qcaf108

**Published:** 2025-09-16

**Authors:** Christian Ovesen, Matthew Dodd, Matthew Ryan, Tim Clayton, Linda Sharples, Divaka Perera

**Affiliations:** Department of Medical Statistics, London School of Hygiene and Tropical Medicine, Keppel Street, London WC1E 7HT, UK; Department of Medical Statistics, London School of Hygiene and Tropical Medicine, Keppel Street, London WC1E 7HT, UK; School of Cardiovascular and Metabolic Medicine and Sciences, British Heart Foundation Centre of Research Excellence, King’s College London, St Thomas’ Hospital, Westminster Bridge Road, London SE1 7EH, UK; Cardiovascular Division, Guy’s and St Thomas’ NHS Foundation Trust, Westminster Bridge Road, London SE1 7EH, UK; Department of Medical Statistics, London School of Hygiene and Tropical Medicine, Keppel Street, London WC1E 7HT, UK; Department of Medical Statistics, London School of Hygiene and Tropical Medicine, Keppel Street, London WC1E 7HT, UK; School of Cardiovascular and Metabolic Medicine and Sciences, British Heart Foundation Centre of Research Excellence, King’s College London, St Thomas’ Hospital, Westminster Bridge Road, London SE1 7EH, UK; Cardiovascular Division, Guy’s and St Thomas’ NHS Foundation Trust, Westminster Bridge Road, London SE1 7EH, UK

**Keywords:** Randomized trials, Percutaneous coronary intervention, Ischaemic cardiomyopathy

## Abstract

**Aims:**

Randomized trials have failed to show a consistent benefit of revascularization in patients with ischaemic cardiomyopathy. This study aimed to investigate whether a participant’s baseline risk introduces heterogeneity in the treatment effect of percutaneous coronary intervention (PCI).

**Methods and results:**

The REVIVED-BCIS2 trial randomised 700 participants with ischaemic cardiomyopathy to PCI plus optimal medical therapy (OMT) vs. OMT alone, 263 (37.6%) of whom experienced the primary outcome (all-cause mortality or hospitalization due to heart failure) during a median follow-up of 41 months. Pre-randomization data were used to develop a prediction model for the primary outcome, and evidence for heterogeneity of the trial intervention effect was investigated. The prediction model included 12 predictors and showed acceptable discrimination (C statistic: 0.69, 95% CI 0.66–0.72) and calibration (calibration slope: 0.79, 95% CI 0.64–0.95). Overall, there was no statistical evidence that the baseline risk score modified the trial intervention effect (*p_interaction_* = 0.21) on the primary outcome. For the secondary outcome of cardiovascular death or hospitalization due to heart failure, weak evidence emerged that the baseline risk modified the trial intervention effect (*p_interaction_* = 0.044). Participants with lower baseline risk trended towards a greater benefit from PCI compared with participants with higher risk scores.

**Conclusion:**

Baseline risk did not influence the effect of revascularization in preventing all-cause mortality or hospitalization due to heart failure. A trend was observed that lower baseline risk might be associated with more benefit of revascularization in preventing cardiovascular death or hospitalization due to heart failure.

**Clinicaltrials.gov identifier:**

NCT01920048

## Graphical Abstract

**Graphical Abstract:**
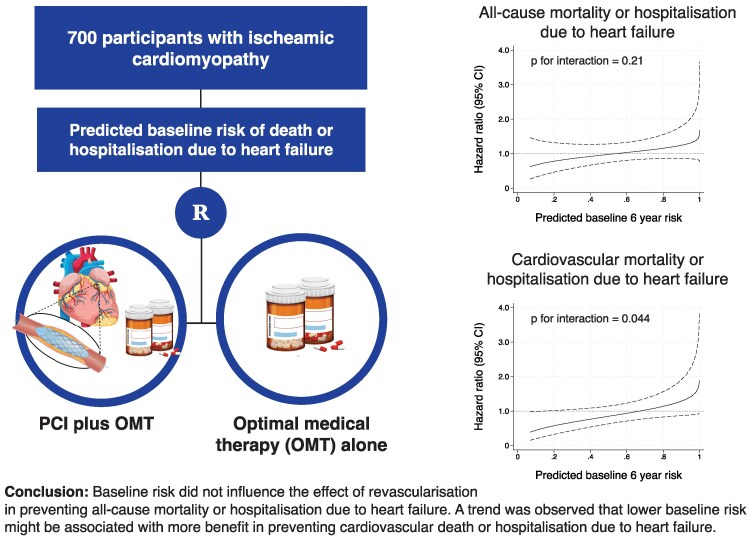

Key Learning Points What is already known

- Current treatment guidelines recommend optimal medical therapy as the mainstay of treatment for patients with heart failure with reduced ejection fraction of ischaemic aetiology, with revascularization to be considered only in selected patients.
- The REVIVED-BCIS2 trial showed no difference in outcome, when randomising participants with ischaemic cardiomyopathy to either revascularization using percutaneous coronary intervention plus optimal medical therapy (OMT) or OMT alone.

What this study adds

- The predicted baseline risk was not a modifier of the treatment effect of revascularization on the outcome of all-cause mortality or hospitalization due to heart failure.
- When considering cardiovascular events in isolation (cardiovascular mortality or hospitalization due to heart failure), lower risk populations may receive more benefit from revascularization.
- The hypothesis that lower risk populations may receive more benefit from revascularization needs confirmation in future studies.

## Introduction

Ischaemic heart disease remains a major contributor to disease burden worldwide and a leading aetiology of heart failure.^1,2^ Current treatment guidelines recommend optimal medical therapy (OMT) as the mainstay of treatment for patients with heart failure with reduced ejection fraction of ischaemic aetiology (ischaemic left ventricular dysfunction), with revascularization to be considered only in selected patients.^3,4^ If revascularization is considered, coronary bypass surgery is the preferred method, based on reductions in all-cause mortality and cardiovascular hospitalization observed in extended follow-up of the Surgical Treatment for Ischaemic Heart Failure (STICH) trial.^5,6^ Until the publication of the Revascularisation for Ischaemic Ventricular Dysfunction [British Cardiovascular Intervention Society (BCIS)] 2 (REVIVED-BCIS2) trial,^7^ the use of percutaneous coronary intervention (PCI) had never been tested against a relevant comparator in a randomised trial of participants with ischaemic cardiomyopathy. In REVIVED-BCIS2, participants with ischaemic cardiomyopathy, extensive coronary artery disease, and demonstratable viable myocardium were randomised to either revascularization using PCI plus OMT or OMT alone.^7,8^ The primary outcome was a composite of all-cause mortality or hospitalization for heart failure. The trial showed no difference in hazard of the primary outcome between treatment groups [hazard ratio (HR) 0.99, 95% CI 0.78–1.27].^7^

While the primary analysis of REVIVED-BCIS2 excluded an overall benefit across the entire trial population, a question that has since been posed is whether there are specific subgroups that may still benefit from revascularization. Heterogeneity of treatment effect between specific subsets of participants has also been demonstrated in other studies of revascularization.^5^ Although such heterogeneity of effect is often characterized by individual patient characteristics (such as age, sex, or specific comorbid diagnoses), the importance of these effects is speculative unless backed by a plausible biological mechanism. As opposed to subgroups formed by individual patient characteristics, a patient’s estimated combined underlying risk of experiencing a negative outcome based on their pre-intervention characteristics can be considered a more credible modifier of treatment effect. This is particularly relevant to interventional studies where the treatment carries a degree of procedure-related risk. Benefit may be mitigated in a low-risk patient who will have good outcome regardless of therapy or conversely may be reduced in high-risk patients who are at greater risk of periprocedural harm, negating the benefit of treatment.

The aims of this prespecified analysis of the REVIVED-BCIS2 trial were to (i) develop a model to predict a participant’s baseline risk of all-cause death or hospitalization due to heart failure, using data from the REVIVED-BCIS2 trial and (ii) investigate whether the predicted baseline risk modified the effect of the trial intervention, with a hypothesis that patients at higher risk would derive greater benefit.

## Methods

This analysis was prespecified in the REVIVED-BCIS2 statistical analysis plan.^7^ The Transparent Reporting of a multivariable prediction model for Individual Prognosis or Diagnosis (TRIPOD) statement^9^ was used to guide the conduct and reporting of this study.

### Participants and trial intervention

The primary results and methodological design of the REVIVED-BCIS2 trial have previously been published.^7,8^ Briefly, REVIVED-BCIS2 was a prospective, multi-center, open-label, randomised trial recruiting participants with ischaemic left ventricular dysfunction. From August 2013 to March 2020, 700 participants were randomised in 40 UK hospitals. Last follow-up was conducted in March 2022. Participants were eligible for REVIVED-BCIS2 if they had (i) left ventricular ejection fraction (EF) ≤35% (assessed by echocardiography or magnetic resonance imaging), (ii) extensive coronary artery disease (defined as a BCIS Jeopardy Score ≥6), and (iii) viability in at least four dysfunctional myocardial segments accessible for revascularization using PCI. Main exclusion criteria were (i) myocardial infarction within 4 weeks prior to randomization, (ii) acute decompensated heart failure (requiring either ionotropic or mechanical circulatory/ventilatory support) or sustained ventricular arrythmia within 72 h prior to randomization, (iii) valve disease requiring imminent intervention, or (iv) contraindication to PCI.

Eligible patients were consented and randomly allocated (1:1) via a web-based application to either revascularization using PCI or no revascularization. Optimal medical therapy was offered to both trial arms and optimization of the medical therapy continued throughout the trial in accordance with trial-specific guidance which was updated regularly over the duration of the trial. Percutaneous coronary intervention was performed in accordance with local protocols. Angioplasty was generally attempted on all lesions in major proximal coronary vessels subtending viable myocardium. Due to the nature of the intervention, participants and care providers were not blinded to allocated treatment.

### Outcome

The primary outcome was a composite of all-cause death or hospitalization due to heart failure. The follow-up period started at randomization, and participants were monitored for the occurrence of the primary outcome throughout the follow-up period. The key secondary outcome was the composite of cardiovascular death and hospitalization for heart failure. Participants were followed for at least 2 years. Events were adjudicated by an independent clinical events committee blinded to treatment assignment.

### Candidate predictors to predict baseline risk

Data from all 700 participants enrolled in REVIVED-BCIS2 were included in this analysis. All baseline variables in the trial database recorded before randomization were considered as candidate predictors for the baseline risk prediction model if they had a biologically and clinically plausible association with the primary outcome. A candidate predictor was dropped if regarded fully represented by another predictor. Core-laboratory adjudicated left ventricular EF was included rather than the site reported EF. Based on subject-matter knowledge provided by clinical experts age at baseline, left ventricular EF and the BCIS Jeopardy score were forced into the prediction model (not subject to selection during the model building phase). Age and left ventricular EF are generally recognised to be important prognostic predictors in patients with ischaemic left ventricular dysfunction.^10–12^ In addition, in the STICH trial, age was shown to be an important modifier of treatment effect of revascularization.^13^ The burden of coronary artery disease (which in the REVIVED-BCIS2 trial was quantified by the BCIS Jeopardy Score^8,14^) has in the SYNTAX II score been shown to be an important prognostic predictor among patients undergoing revascularization using PCI.^15^ No clinically relevant interaction terms between the baseline variables and the outcome were anticipated. Therefore, given the size of the dataset (700 participants), no interaction effects were investigated when building the prediction model. Trial intervention was not included in the prediction model as it was not associated with the outcomes in the REVIVED-BCIS2 trial. No formal sample size calculation for this analysis was undertaken.

### Statistical analysis

Data were presented as frequencies (percentage), mean (standard deviation) or median (interquartile range) as appropriate. Univariable Cox proportional hazards models were used to explore the association between each baseline predictor and the primary outcome (all-cause death or hospitalization due to heart failure) in complete case analyses. Before development of the multivariable prediction model, multiple imputations with chained equations (MICEs) were used to impute missing data under the missing-at-random assumption (full details provided in supplementary material online, *Methods S1*). Ten imputed datasets were created. A candidate multivariable Cox proportional hazards prediction model was developed based on a forward stepwise model building approach as prespecified in the REVIVED-BCIS2 statistical analysis plan.^7^ The dependent variable was the primary outcome (all-cause death or hospitalization due to heart failure). See the supplementary material online, *Methods S2* for details on the model-building procedures.

Model performance was assessed using prediction error (Brier score at 2, 4, and 6 years of follow-up scaled by the overall Kaplan–Meier estimator), model discrimination (Harrells C and Gönen and Hellers K) and calibration (calibration slope and calibration plots at 2, 4, and 6 years of follow-up). Optimism in model performance was corrected for using Bootstrapping (see supplementary material online, *Methods S3*).

To investigate if the predicted baseline risk of the primary outcome modified the effect of the trial intervention, the risk score was calculated for each participant using the final prediction model. Before the calculation of the risk score, the missing data were imputed using an imputation model that ensured compatibility with the analysis model (see supplementary material online, *Methods S4*). To assess whether the effect of the intervention (PCI plus OMT vs. OMT alone) on the primary outcome was modified by baseline risk, a Cox proportional hazards model including predicted baseline risk score (continuous), trial allocation and their interaction were fitted to each imputed dataset. A second analysis was conducted where participants were classified into three groups according to their predicted baseline risk (low, medium, and high risk) so that there were approximately equal numbers of outcome events within each group. Cox proportional hazards models including risk group, trial treatment, and their interaction were fitted to each imputed dataset. In both cases, model coefficients (and variances) were combined on the log-scale using Rubin’s rules before being transformed to the hazard ratio scale. Evidence for heterogeneity was assessed based on the interaction terms. The analyses listed above were repeated with the secondary outcome restricted to cardiovascular death or hospitalization due to heart failure.

As traditional stepwise model-building can be prone to overfitting and consequently poorer external model calibration, a sensitivity analysis was performed using the Least Absolute Shrinkage and Selection Operator (LASSO) to build a candidate prediction model (see supplementary material online, *Methods S5*). For the LASSO model, the evaluation of the model performance, and the assessment of the modifying properties on the trial intervention effect was performed as listed above. All statistical analyses were conducted on an intention-to-treat basis using STATA 18.0 (StataCorp, TX, USA).

## Results

Seven hundred participants were included in the trial database. The baseline characteristics (and missingness in each variable) are presented in supplementary material online, *Table S1*. Three hundred forty-seven (49.6%) participants were randomised to PCI plus OMT and 353 (50.4%) to OMT alone. The mean (standard deviation) age at randomization was 69.4 (9.1) years, and 614 (87.7%) were males. Five hundred and thirteen (73.8%) had a New York Heart Association (NYHA) heart failure classification of I or II. Median [interquartile range (IQR)] left ventricular EF was 32.0% (24.4–38.3%) and median (IQR) BCIS Jeopardy score at baseline was 10.0 (8.0–12.0). The median (IQR) number of lesions to be treated was 2.0 (2.0–3.0). The overall median (IQR) follow-up time was 41 (28–60) months. Six hundred ninety-three (99.0%) were followed for the protocolized minimum of 2 years. Two hundred sixty-three (37.6%) participants experienced a first primary outcome event. The first event was all-cause death for 158 (60.1%) participants and hospitalization due to heart failure for 105 (39.9%) participants. In participants who died, the cause of death was cardiovascular in 164 (72.9%) and non-cardiovascular in 61 (27.1%).

### Prediction models

The univariable association between hazard of the primary outcome and each candidate predictor is presented in supplementary material online, *Table S2*. The final stepwise model consisted of 12 predictors (*Table 1*). Adjusted for the other variables in the model, there was no evidence (*P* > 0.1) that the age at randomization, BCIS Jeopardy score or left ventricular EF were associated with the hazard of the primary outcome. Higher resting heart rate (per 5 beat/minute: HR 0.94, 95% CI 0.89–0.99) and beta-blocker use at baseline (HR 0.56, 95% CI 0.37–0.85) were associated with decreased hazard of the primary outcome. Peripheral vascular disease (HR 1.70, 95% CI 1.25–2.32), NYHA grade III or IV (compared with I or II) (HR 1.44, 95% CI 1.11–1.88), hospitalization due to heart failure within the last 2 years prior to randomization (HR 1.41, 95% CI 1.08–1.83), and the use of diuretics at baseline (HR 1.39, 95% CI 1.04–1.86) were associated with increased hazard of the primary outcome. In addition, increasing baseline levels of N-terminal fragment of the prohormone brain-type natriuretic peptide (NT-proBNP) [per log(ng/L): HR 1.47, 95% CI 1.30–1.67], haemoglobin A1c (per 5 mmol/mol: HR 1.06, 95% CI 1.02–1.11), and serum creatinine [per log(µmol/L): HR 1.69, 95% CI 1.20–2.39] were also associated with increasing hazard of the primary outcome.

**Table 1:** Final prediction model for the primary outcome (*n* = 700)

Predictor	β	HR	95% CI
Age at randomization (per 5 year)	0.059	1.06	0.98 to 1.14
BCIS Jeopardy score (per 2 point increase)^a^	0.041	1.04	0.94 to 1.15
Left ventricular EF (per 5% increase)	0.045	1.05	0.97 to 1.12
Heart rate (per 5 beats/minute increase)	−0.067	0.94	0.89 to 0.99
Peripheral vascular disease	0.533	1.70	1.25 to 2.32
New York Heart Association Classification			
Grade I or II	0.000	1.00	Reference
Grade III or IV	0.366	1.44	1.11 to 1.88
Admission due to HF (previous 2 years)	0.341	1.41	1.08 to 1.83
Log of NT-proBNP [per log(ng/L) increase]	0.386	1.47	1.30 to 1.67
Haemoglobin A1c (per 5 mmol/mol increase)	0.062	1.06	1.02 to 1.11
Log of creatinine [per log(µmol/L) increase]	0.525	1.69	1.20 to 2.39
Loop or thiazide diuretics use	0.330	1.39	1.04 to 1.86
Beta-blocker use	−0.576	0.56	0.37 to 0.85

Baseline survival probabilities (Stepwise model): $S_{0}(2)=0.9996$ (2-year), $S_{0}(4)=0.9993$ (4-year), and $S_{0}(6)=0.9987$ (6-year).

β, beta coefficients from the model; BCIS, British Cardiovascular Intervention Society; EF, ejection fraction; HR, hazard ratio; log, natural log-transformation; NT-proBNP, N-terminal fragment of the prohormone brain-type natriuretic peptide.

^a^Variable centered at 2 points.

When compared with the overall rate of the primary outcome at the given point in time, the model yielded a moderately improved optimism-corrected prediction error (scaled Brier score) when assessed after 2 years [9% (95% CI 3% to 16%)], 4 years [10% (95% CI 3% to 18%)], and 6 years [10% (95% CI −2% to 21%)] of follow-up. For the full follow-up period, the optimism-corrected Harrell´s C statistic was 0.69 (95% CI 0.66–0.72), and the optimism-corrected Gönen and Hellers K statistic was 0.70 (95% CI 0.67–0.72). When recalculating the optimism-corrected Harrell’s C and Gönen and Hellers K statistic after 2 and 4 years of follow-up, these were comparable to the statistics for the full follow-up period (see supplementary material online, *Table S3*). The optimism-corrected calibration slope was 0.79 (95% CI 0.64–0.95). The apparent calibration plots after 2, 4, and 6 years of follow-up are available in supplementary material online, *Figure S1*. A calculator for the risk score and predicted probabilities of the primary outcome after 2, 4, and 6 years is included in supplementary material online, *File S1*.

### Subgroup analysis

**Figure 1:**
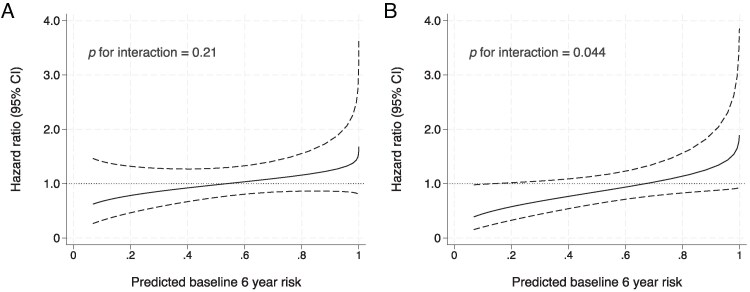
Intervention effect for different predicted baseline risks of the primary outcome after 6 years of follow-up. The intervention effect (solid line) and 95% confidence intervals (dashed lines) of percutaneous coronary intervention plus optimal medical therapy vs. optimal medical therapy alone. (*A*) Represents the outcome all-cause mortality or hospitalization due to heart failure and (*B*) represent the outcome cardiovascular mortality or hospitalization due to heart failure. A hazard ratio <1.00 favour percutaneous coronary intervention plus optimal medical therapy.

The distributions of predicted risk scores and 6-year event probabilities are presented in supplementary material online, *Figure S2*. The estimated intervention effect (PCI plus OMT vs. OMT alone) across the spectrum of model-based risk scores (modelled as continuous variable) is presented in *Figure 1*. There was no statistical evidence that the risk scores modified the effect of the trial intervention (*P* for interaction = 0.21).

**Figure 2:**
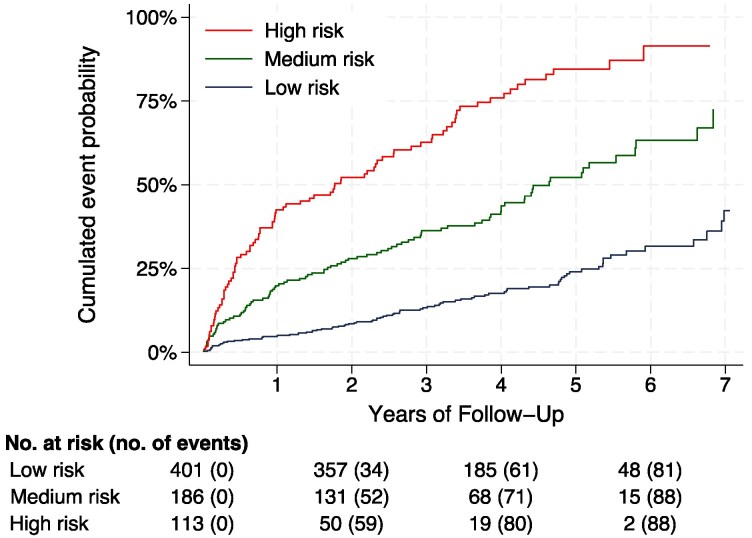
Event curves of all-cause mortality or hospitalization due to heart failure among risk groups. Participants are separated into three risk strata based on their predicted baseline risk of all-cause mortality or hospitalization due to heart failure—a low risk group, a medium risk group and a high risk group. A participant’s risk score is averaged over the imputed datasets. Below the figures, the number at risk and the cumulative events are presented.

The cumulative probability of the primary outcome within each risk group is presented in *Figure 2* (and separated out by trial arm in supplementary material online, *Figure S3*). The low-risk group had a model-based predicted 6-year event probability < 50.8% (risk score <6.3), the medium risk group with a predicted 6-year event probability 50.8–76.0% (risk score ≥6.3 and <7.0), and the high-risk group with a predicted 6-year event probability >76.0% (risk score >7.0). There was no statistical evidence of heterogeneity in treatment effect between risk groups (*P* for interaction = 0.25, *Table 2*). When participants were divided into five risk groups with an approximate equal number of outcome events in each group (see supplementary material online, *Table S4*), the results were consistent with those presented above.

**Table 2:** Effect of trial intervention stratified by baseline risk of primary outcome

Risk groups [6-year event risk]^a^	Risk difference, percentage points (95% CI)^b^		HR (95% CI)^c^	*P* for interaction
	At 2 years	At 4 years		
All-cause mortality or hospitalization due to heart failure				
Low risk [<50.8%]	0.6 (−5.3 to 6.5)	−0.3 (−9.3 to 8.6)	0.83 (0.53 to 1.29)	0.25
Medium risk [50.8 to 76.0%]	−3.4 (−20.3 to 13.4)	6.8 (−13.5 to 27.2)	1.03 (0.65 to 1.61)	
High risk [>76.0%]	19.5 (−1.8 to 40.8)	11.4 (−10.8 to 33.6)	1.46 (0.90 to 2.37)	
Cardiovascular mortality or hospitalization due to heart failure				
Low risk [<50.1%]	0.8 (−5.5 to 7.0)	−4.1 (−12.7 to 4.6)	0.70 (0.42 to 1.16)	0.16
Medium risk [50.1 to 76.0%]	−1.7 (−16.7 to 13.4)	−1.0 (−19.4 to 17.3)	0.95 (0.57 to 1.58)	
High risk [>76.0%]	17.1 (−5.2 to 39.3)	13.2 (−9.9 to 36.4)	1.42 (0.87 to 2.31)	

Data in table are based on 10 imputed datasets. Risk groups are formed to allow approximate equal number of events in each group.

CI, confidence interval; HR, hazard ratio; No., number.

^a^Numbers in square brackets are the predicted 6-year probability of the primary outcome used as cut-points to form the risk groups.

^b^Absolute risk difference based on the Kaplan–Meier estimator [negative values favour percutaneous coronary intervention (PCI)] plus optimal medical therapy (OMT). The 95% confidence intervals are based on 500 bootstrap replications in each imputed dataset.

^c^Hazard ratios <1.00 favour PCI plus OMT.

For the secondary outcome of cardiovascular death or hospitalization due to heart failure, risk scores (when modelled as a continuous term) modified the effect of the trial intervention (*P* for interaction = 0.044, *Figure 1*), with a visual trend towards participants with lower predicted baseline risk experiencing a greater benefit from PCI compared with participants with higher baseline risk scores. When stratified into risk groups (low, medium, and high), no statistical evidence was observed for heterogeneity (*P* for interaction = 0.16, *Table 2*).

### Sensitivity analysis using least absolute shrinkage and selection operator

As described above, a sensitivity analysis using LASSO was conducted to supplement the pre-specified stepwise model-building. The LASSO model consisted of 21 predictors (see supplementary material online, *Table S5*). The performance statistics of the LASSO model were comparable to the stepwise model presented above with optimism-corrected Harrell´s C statistic of 0.70 (95% CI 0.66–0.73) and optimism-corrected calibration slope of 1.04 (95% CI 0.84–1.25) (see supplementary material online, *Table S6*). The apparent calibration plots after 2, 4, and 6 years of follow-up are available in supplementary material online, *Figure S4*. When the LASSO prediction model was used to estimate the intervention effect (PCI plus OMT vs. OMT alone) across the spectrum of model-based risk scores, results were comparable to the results obtained from the stepwise model above with no statistical evidence that the risk scores modified the effect of the trial intervention on the primary outcome of all-cause mortality or hospitalization due to heart failure (*P* for interaction = 0.65) (see supplementary material online, *Figure S5* and *S6*, and supplementary material online, *Table S7*).

## Discussion

We constructed a prediction model using predictors that are readily available for patients with ischaemic cardiomyopathy in a clinical setting. The model showed acceptable performance (prediction error, discrimination, and calibration). No statistical evidence was found that baseline risk modified the effect of the trial intervention on the primary outcome (all-cause mortality and hospitalization due to heart failure). When restricting the outcome to cardiovascular death or hospitalization due to heart failure, statistical evidence that the baseline risk modified the trial intervention effect was observed. A trend was observed that participants with lower baseline risk scores experienced a greater benefit from revascularization compared with participants with higher baseline risk. The reported results were robust to a change in the model-building strategy as the LASSO model yielded similar results.

A limitation of this analysis was the fact that the prediction models used to stratify the participants into risk groups were developed in the same cohort, although the risk groups were based on the statistical principle of equal events per subgroup, rather than on choosing thresholds that maximise differences in outcomes between subgroups within this sample. Even though a modern approach to internal validation was utilised, it remains to be seen, if the model is valid and performs well in external populations. The consequence is that the prediction model may not be generalisable to external samples that differ from the derivation cohort, and external validity should be investigated before it is used in other populations. Since the model has been shown to be internally valid, it should perform acceptably in samples similar to the derivation cohort. Moreover, the lack of external validation does not invalidate the model’s application when investigating heterogeneity of treatment effect in this study. Another important limitation of this analysis is the low statistical power. Subdividing a sample from a clinical trial often results in insufficient statistical power to detect even substantial subgroup effects.^16^ Consequently, it is possible that even if the baseline risk of the primary outcome was an important modifier of the intervention effect, the REVIVED-BCIS2 trial sample would not have enough data power to detect it. Studies using larger databases are required to confirm these results.

Selection of predictors for the models was largely data driven, which risks omitting important but infrequently observed predictors, although an effort was made to incorporate clinical knowledge into the model-building. The REVIVED-BCIS2 trial statistical analysis plan^7^ prespecified the use of forward stepwise selection with a selection criterion of a *P* < 0.05 in order to employ a simple and well-known approach. The forward stepwise selection approach has been criticised for producing extreme estimates of the regression coefficients and generating inflated type 1 errors. Therefore, we also produced a model based on the LASSO, a more contemporary approach to building prediction models which allows model refinement via a tuning parameter. The LASSO is expected to produce enhanced external validity of the models, although it had similar predictive performance to the primary analysis in this study.

Our risk prediction model was effective in predicting subsequent risk. It is surprising, however, that factors typically considered to be strong predictors of outcome were not significant predictors in our model, specifically age, the extent of coronary artery disease and left ventricular ejection fraction. It must be remembered, however, that the REVIVED-BCIS2 trial only included participants with the most severe left ventricular impairment and the most severe coronary disease—and hence the trial population may lack the resolution to detect the impact of variation in these factors across the whole range.

The use of the data from randomised clinical trials to develop clinical prediction models is attractive, due to the prospective data collection and well-defined variables generated through a methodologically rigorous process. However, there are limitations with this approach. Even though the REVIVED-BCIS2 trial was relatively large, the sample size was only sufficient to detect strong associations between clinical predictors and outcome. Also, important interactions could have remained undetected due to low statistical power.^17^ A general limitation when using randomised clinical trial data for the purpose of prediction model development is the relatively strict selection criteria often imposed.^18^ This can result in homogeneous samples, making it more difficult to identify the effect of the predictor variables (variation is needed to see the effect of predictors). The external validity of the models can also be affected by the strict selection criteria, as trial participants might be different from the target population in important aspects.

In our secondary analysis focussing on cardiovascular death and hospitalization for heart failure, the heterogeneity of treatment effect reached statistical significance when risk was treated as a continuous variable. This observation seems intuitive, as both cardiovascular death and hospitalization for heart failure are more likely to be modifiable by PCI than non-cardiovascular death. The observation should be considered in the context of the wider literature about the influence of baseline risk on the effectiveness of coronary revascularization. Even though both the EuroSCORE-2 and the Society of Thoracic Surgeons score (STS) have been shown to predict 30-day mortality in the STICH population, whether the predicted risk of poor outcome at baseline modifies the treatment effect in the STICH trial, has (to our knowledge) never been tested.^19^ Several secondary analyses pertaining to individual specific baseline characteristics have been reported. Consistent with the results of our analyses, factors generally considered to be consistent with lower risk (including lower age, higher 6-minute walk distance, and higher physical ability assessment) have been associated with greater treatment benefit of revascularization.^13,20^

The Synergy Between Percutaneous Coronary Intervention with TAXUS and Cardiac Surgery (SYNTAX) II score is an 8-component risk score, which has been shown effective in predicting 4-year mortality in patients with complex coronary artery disease undergoing either PCI or coronary artery bypass grafting.^15^ Even though the population and level of baseline risk in the SYNTAX trial were fundamentally different from that of the REVIVED-BCIS2 trial, factors like age, LVEF, creatinine, extent of coronary atherosclerotic disease, and peripheral vascular disease are common predictors included in both risk scores. Based on the SYNTAX II score, the benefit of coronary artery bypass grafting over PCI was more pronounced in those with higher risk over the course of follow-up.^15^ This aligns with our finding that revascularization using PCI was associated with unfavourable prognosis in higher risk populations. Contradictory to the findings of the SYNTAX II score, our findings suggest a better prognosis for patients with a higher risk prediction when managed with optimal medical treatment compared with revascularization. This discrepancy between recent trials and past trials could indicate that modern optimal medical therapy has alleviated some of the benefit of revascularization observed in previous trials. This notion was supported by a recent study showing that participants randomised to optimal medical therapy in the REVIVED-BCIS2 trial have a much better prognosis compared with those randomised to optimal medical therapy in the STICH trial.^21^ Similarly, it has been shown that the prognosis after PCI in patients with severe coronary artery disease has improved dramatically since the publication of the SYNTAX trial.^22^

The existence of a subpopulation, who will benefit from revascularization, has previously been hypothesised. This might be a population with sufficient room for the efficacy of treatment to overcome procedural risk, but before the risk of adverse outcomes becomes insurmountable or the natural history of the condition becomes driven more by the phenotype of heart failure than new coronary events. This hypothesis also mirrors the conclusion of a subgroup analysis based on a smaller subset of the ISCHEMIA population (∼8%) with mid-range ejection fraction (35–45%) or a history of heart failure.^23^ It was concluded that this population with mid-range ejection fraction or a history of heart failure trended towards more benefit from an invasive revascularization treatment strategy compared with a more conservative treatment approach. This effect was primarily driven by a large treatment effect among the participants with both heart failure and a mid-range ejection fraction, although this group constituted only ∼0.5% (28 participants) of the ISCHEMIA trial population.^23^

The hypothesis generated by this study that participants with ischaemic left ventricular dysfunction and a low baseline risk may experience more benefit from revascularization using PCI as compared with optimal medical management is potentially important and merits further research in future prospective studies. The apparent inconsistency between observational studies that found a staggering reduction in mortality related to revascularization and the neutral results of REVIVED-BCIS2 may be due to patient selection bias.^7,24^ That is, patients having revascularization in observational studies might generally have a lower baseline risk profile compared with those managed using optimal medical therapy. Furthermore, the discordance between the results of REVIVED-BCIS2 and STICH might be explained by differences in the risk profiles of the enrolled populations, although the different mechanisms of revascularization and progressive improvements in medical therapy over time are reasonable alternative hypotheses.

## Conclusion

Among participants with ischaemic left ventricular dysfunction, the predicted baseline risk could not be shown to modify the intervention effect of revascularization on the outcome of all-cause mortality or hospitalization due to heart failure. A trend was observed for lower risk populations receiving more benefit from revascularization on the outcome of cardiovascular mortality or hospitalization due to heart failure. These results are hypothesis generating and need confirmation in larger, prospective, patient cohorts.

## Supplementary Material

qcaf108_Supplementary_Data

## Data Availability

The data underlying this article can be shared on reasonable request by contacting the corresponding author.
