# Supplementary material for: Impact of baseline risk of death or hospitalization on effectiveness of revascularization in patients with ischaemic left ventricular dysfunction—a prespecified analysis of REVIVED-BCIS2
Source: Eur Heart J Qual Care Clin Outcomes. 2025 Sep 16;11(8):1440–7. doi: 10.1093/ehjqcco/qcaf108 (PMC12714387; doi:10.1093/ehjqcco/qcaf108)
Supplement: qcaf108_Supplementary_Data [file qcaf108_supplementary_data.zip › Supplementary file 1.1 - README_REVIVED_calculator.pdf]

Supplementary file 1: REVIVED score calculator

The calculator calculates the risk score and predicted probabilities of all-cause death or hospitalisation due to heart failure within 2, 4 and 6 years.

The calculator has been included as a macro-based and a non-macro-based Excel file. When using the macro-based calculator, macros should be activated for the document to function.

Calculation:
The calculator uses the coefficients from the score to calculate the combined risk score. The baseline survival function and the combined risk score are used to calculate the probability of the outcome.

The REVIVED score

Predictor	β
Age at randomisation (per 5 year)	0.059
BCIS Jeopardy score (per 2 point increase)*	0.041
Left ventricular ejection fraction (per 5% increase)	0.045
Heart rate (per 5 beats/minute increase)	-0.067
Peripheral vascular disease	0.533
New York Heart Association Classification	
Grade I or II	0.000
Grade III or IV	0.366
Admission due to HF (previous 2 years)	0.341
Log of NT-proBNP (per log[ng/L] increase)	0.386
Haemoglobin A1c (per 5 mmol/mol increase)	0.062
Log of creatinine (per log[µmol/L] increase)	0.525
Loop or thiazide diuretics use	0.330
Beta-blocker use	-0.576

* Centered at 2 points

The score is calculated as follows:

$$Risk\ score = 0.059 * \left(\frac{age}{5}\right) + 0.041 * \left(\frac{(Jeopardy\ score - 2)}{2}\right) + 0.045 * \left(\frac{LVEF}{5}\right) + (-0.067) * \left(\frac{HR}{5}\right)$$
$$+ 0.533 * (PVD) + 0.366 * (NYHA\ grade\ III\ or\ IV) + 0.341$$
$$* (admission\ due\ to\ heart\ failure) + \ 0.386 * \ln(NTproBNP) + 0.062 * \left(\frac{HbA1c}{5}\right)$$
$$+ 0.525 * \ln(creatinine) + 0.330 * (diuretics) + (-0.576) * (betablocker)$$

Where: LVEF – Left ventricular ejection fraction, HR – heart rate, PVD – Peripheral vascular disease, NYHA – New York Heart Association

Categorical variables are entered as 1 if present and 0 if not present

The risk predictions are calculated using baseline survival probabilities: $S_0(2) = 0.9996$ (2-year), $S_0(4) = 0.9993$ (4-year) and $S_0(6) = 0.9987$ (6-year)

Consequently, to predict 2-year event probability from the risk score:

$$Event\ probability\ (2\text{-}year) = 1 - 0.9996^{e^{risk\ score}}$$

In a similar manner the event probabilities after 4 and 6 years can be calculated as:

$$Event\ probability\ (4\text{-}year) = 1 - 0.9993^{e^{risk\ score}}$$
$$Event\ probability\ (6\text{-}year) = 1 - 0.9987^{e^{risk\ score}}$$
