# Supplementary material for: Impact of baseline risk of death or hospitalization on effectiveness of revascularization in patients with ischaemic left ventricular dysfunction—a prespecified analysis of REVIVED-BCIS2
Source: Eur Heart J Qual Care Clin Outcomes. 2025 Sep 16;11(8):1440–7. doi: 10.1093/ehjqcco/qcaf108 (PMC12714387; doi:10.1093/ehjqcco/qcaf108)
Supplement: qcaf108_Supplementary_Data [file qcaf108_supplementary_data.zip › supplementary_prediction_revived_v.2.4_28.6.25.pdf]

SUPPLEMENTARY METHODOLOGY

Supplementary methods 1: Multiple imputation model before prediction model development

All imputation models included all the potential predictors (except for the variable being imputed) including the trial allocation, the Nelson-Aalen estimator of the cumulative hazard, and an event indicator for the outcome variable.[1,2] The imputation models were constructed to be as rich as possible to ensure that the final prediction model was nested within it. It was originally decided to impute missing data separately in each of the two trial allocation groups (PCI plus OMT versus OMT alone), however, this resulted in convergence issues. Instead, trial allocation was included as an auxiliary variable in all imputation models.

Missing values in continuous variables with an approximate normal distribution *(log-transformed mean arterial pressure, heart rate, log-transformed haemoglobin, and log-transformed creatinine)* were imputed using linear regression, missing values in binary variables *(atrial fibrillation on baseline electrocardiogram (ECG), NYHA heart failure classification [dichotomised], family history of premature ischaemic heart disease, hypertension, ACE-inhibitor use, angiotensin II receptor blocker use, aldosterone antagonist use, loop or thiazide diuretics use, digoxin use, amiodarone use)* were imputed using logistic regression, missing values in un-ordered categorical variables *(ethnicity and antiplatelet use)* were imputed using multinomial model, and ordered categorical *(Canadian Cardiovascular Society angina grade and body mass index [categorical])* using ordinal logistic regression. Non-normally distributed continuous variables *(number of atherosclerotic lesions to be treated, BCIS Jeopardy score, left ventricular EF, total cholesterol, low-density lipoprotein, high-density lipoprotein, triglyceride, log-transformed NT-proBNP, and HbA1c)* were imputed using predictive mean matching. The donor for predictive mean matching was selected from a pool of the 5 closest participants.

The algorithm was set to run 20 cycles as burn-in. 10 imputed datasets were created. The distributions of the imputed values in the imputed datasets were compared to the observed data to detect outliers or problems in the imputation model.

Multiple imputation was conducted in the 'mi' package of STATA 18.0 (StataCorp, TX, USA).

Supplementary methods 2: Model building using stepwise selection of covariates in imputed data sets

A forward stepwise selection was used with a selection criterion of $p<0.05$ for a predictor to be included in the model. The process was complicated by the fact that multiple imputation was used to handle missing data. A two-step procedure was applied to perform the stepwise model selection in the imputed dataset. Previously, it has been shown that an acceptable approach to automated model selection in imputed datasets is to perform the model selection on the stacked datasets with a fixed weight applied to cases.[3] Consequently, a stacked dataset was generated with $m \times N$ cases with m being the number of imputed datasets and N being the number of participants in the original dataset. A fixed weight of $w = \frac{1}{m}$ was applied to each observation.

The stepwise forward selection was conducted as follows[3-5]: (1) The automated variable selection procedure started with the null-model. (2) All the candidate predictors were iteratively entered in the model (generating a series of univariable models), and the most statistically significant candidate predictor (based on a weighted partial likelihood ratio test) among all the univariable models was added to the prediction model. (3) Predictors from the remaining pool of candidates were added iteratively to the model from the previous step and the most statistically significant predictor retained. (4) This process was continued, until no candidate predictor achieved a *p*-value below 0.05 when added to the model. When the stepwise model selection was completed, the final set of predictors was fitted in each of the m imputed datasets and combined using Rubin's rule to obtain the final prediction model.

The modelling assumptions for the Cox proportional hazards model were evaluated in each of the first 6 imputed datasets. The proportional hazards assumption and linearity of continuous predictors were examined using plots of scaled Schoenfeld residuals and Martingale residuals respectively as described for the univariable Cox proportional hazards.

Supplementary methods 3: Internal validation procedure

To obtain optimism adjusted performance statistics for the two prediction models, the optimism was estimated based on an internal validation procedure. This was performed in accordance with the methodology suggested by Harrell et al. [6] and Steyerberg.[5]

For this procedure, a single imputed dataset (from the multiple imputation procedure described above) was used.

1. A bootstrap resample from the imputed dataset was drawn.
2. The model-building procedures described above (although only running in a single dataset) were applied to the bootstrap resample yielding a candidate prediction model.
3. The apparent performance statistics of the model were calculated in the bootstrap sample (Brier score at 2, 4 and 6 years of follow-up, model discrimination [Harrells C and Gönen and Hellers K] and calibration slope).
4. The model was then evaluated in the original single imputed dataset, and the performance statistics of the model were calculated.
5. The difference between the performance statistics in the original imputed dataset and the bootstrap sample was interpreted as an estimate of the optimism.
6. Step 1-5 was repeated 500 times to obtain a stable estimate of the optimism.

The optimism was estimated for both the stepwise prediction model and the LASSO prediction model (as described below). The final estimate of the optimism was used to correct the performance statistics of the two models to obtain an optimism-corrected performance.

Supplementary methods 4: Multiple imputation model before the subgroup analysis:

For the subgroup analysis, it was necessary to re-impute the dataset using an imputation model approximately compatible with the planned analysis model, as the original imputation model (used for fitting of the prediction models) was not compatible with a potential interaction effect between the trial intervention and the risk scores. As one or more predictor values of the LASSO model (the stepwise model containing a subset of the predictors in the LASSO model) were missing in the original dataset for 49.3% of the participants, these could not receive a risk score. All predictors included in the LASSO model were included in the imputation model, in addition to the Nelson-Aalen estimator of the cumulative hazard, and an event indicator for the outcome variable. To avoid shrinkage of a potential interaction effect between the trial intervention and the risk score towards zero (if it exists), multiple imputation was performed separately in each treatment group.

Missing data in continuous variables with an approximate normal distribution *(log-transformed mean arterial BP, heart rate, and log-transformed creatinine)* were imputed using linear regression, missing data in binary variables *(NYHA heart failure classification [dichotomised], family history of premature ischaemic heart disease, hypertension, angiotensin II receptor blocker use, loop or thiazide diuretics use)* were imputed using logistic regression, and ordered categorical *(body mass index [categorical])* using ordinal logistic regression. Non-normally distributed continuous variables *(BCIS Jeopardy score, left ventricular EF, total cholesterol, log-transformed NT-proBNP, and HbA1c)* were imputed using predictive mean matching. The donor for predictive mean matching was selected from a pool of the 5 closest participants. The algorithm was set to run 20 cycles as burn-in. 10 imputed datasets were created. Multiple imputation was conducted in the 'mi' package of STATA 18.0 (StataCorp, TX, USA). The distributions of the imputed values in the imputed datasets were compared to the observed data to detect outliers or problems in the imputation model.

Supplementary methods 5: Model building using LASSO in imputed data sets

In a sensitivity analysis the LASSO-Cox proportional hazards model was used to construct an alternative prediction model to the one obtained using stepwise variable selection. As multiple imputation was used to handle missing data (resulting in m imputed datasets), it was problematic to use the classic LASSO approach with cross-validation. If the LASSO was applied to each individual dataset, it would have resulted in m different model choices. In order to overcome this problem, a pragmatic approach suggested by Steyerberg[5] was employed.

The LASSO-Cox model was first fitted independently in each imputed dataset. The selected optimal λ for each model was recorded. The LASSO-Cox model was then fitted to the stacked imputed datasets ($m \times N$ cases) with a fixed weight $\left(w = \frac{1}{m}\right)$ applied to the cases. The LASSO-Cox model was fitted without cross-validation, as the cross-validation has previously been reported to perform poorly in the stacked datasets[5]. The median λ from the individual imputed datasets was used as the penalisation parameter. The penalised and unstandardised coefficients were obtained as final coefficients in the prediction model.

The modelling assumptions were checked by examining the assumptions of the final model in each of the first 6 imputed datasets. The proportional hazards assumption and linearity of continuous predictors were examined using plots of Schoenfeld residuals and Martingale

SUPPLEMENTARY TABLES

Supplementary table 1: Baseline characteristics of the trial population

Supplementary table 1: Baseline characteristics of the trial population

Characteristic	Total population (N=700)	Missing values, n (%)
**Trial allocation**		0 (0%)
Optimal medical therapy alone	353 (50.4%)	
PCI plus optimal medical therapy	347 (49.6%)	
**Baseline general characteristics**		
Age at randomisation (years)	69.4 (9.1) [34.7 to 89.3]	0 (0%)
Male gender	614 (87.7%)	0 (0%)
Body weight (kg)	84.7 (18.2) [40.0 to 154.0]	4 (0.6%)
Height (cm)	172.0 (8.7) [131.0 to 191.0]	3 (0.4%)
Body mass index (kg/m^2)	28.6 (5.5) [14.9 to 51.3]	4 (0.6%)
Ethnicity		4 (0.6%)
Afro-Caribbean	6 (0.9%)	
Asian	49 (7.0%)	
Caucasian	634 (91.1%)	
Mixed	2 (0.3%)	
Other	5 (0.7%)	
**Baseline clinical characteristics**		
Systolic blood pressure (mmHg)	125.3 (20.0) [76.0 to 188.0]	2 (0.3%)
Diastolic blood pressure (mmHg)	71.7 (12.0) [35.0 to 129.0]	2 (0.3%)
Mean arterial blood pressure (mmHg)	89.6 (13.2) [50.7 to 146.0]	2 (0.3%)
Heart rate (beats/minute)	70.0 (12.3) [43.0 to 129.0]	2 (0.3%)
CCS angina grade		3 (0.4)
No angina	464 (66.6%)	
I	143 (20.5%)	
II	75 (10.8%)	
III	14 (2.0%)	
IV	1 (0.1%)	
NYHA heart failure classification		5 (0.7%)
I	126 (18.1%)	
II	387 (55.7%)	
III	172 (24.7%)	
IV	10 (1.4%)	

Left ventricular ejection fraction (%)	32.0 (24.4 to 38.3) [10.3 to 63.8]	160 (22.9%)
Atrial fibrillation on baseline ECG	114 (17.4%)	43 (6.1%)
Angiogram at baseline	690 (98.6%)	0 (0%)
Number of atherosclerotic lesions to be treated	2.0 (2.0 to 3.0) [0.0 to 8.0]	11 (1.6%)
BCIS Jeopardy score	10.0 (8.0 to 12.0) [2.0 to 12.0]	3 (0.4%)
**Cardiovascular risk factors**		
Smoking status		0 (0%)
Never smoker	190 (27.1%)	
Exsmoker	374 (53.4%)	
Current smoker	136 (19.4%)	
Hypertension	391 (55.9%)	1 (0.1%)
Hypercholesterolemia	382 (54.6%)	0 (0%)
Peripheral vascular disease	94 (13.4%)	0 (0%)
Diabetes	289 (41.3%)	0 (0%)
Dialysis treatment	9 (1.3%)	0 (0%)
Family history of premature IHD	250 (36.0%)	5 (0.7)
Previous cerebrovascular disease	84 (12.0%)	0 (0%)
Previous myocardial infarction	372 (53.1%)	0 (0%)
Previous PCI	142 (20.3%)	0 (0%)
Previous CABG	34 (4.9%)	0 (0%)
Hospital admission due to HF (previous 2 years)	233 (33.3%)	0 (0%)
**Baseline biochemistry**		
Haemoglobin (g/L)	135.1 (16.2) [82.0 to 182.0]	19 (2.7%)
Creatinine (μmol/L)	110.9 (54.4) [41.0 to 690.0]	9 (1.3%)
Total cholesterol (mmol/L)	4.0 (1.2) [1.5 to 10.8]	78 (11.1%)
Low-density lipoprotein (mmol/L)	2.0 (0.9) [0.3 to 5.3]	221 (31.6%)
High-density lipoprotein (mmol/L)	1.2 (0.4) [0.1 to 3.5]	140 (20.0%)
Triglyceride (mmol/L)	1.8 (1.1) [0.4 to 7.9]	111 (15.9%)
NT-ProBNP (ng/L)	3144.4 (5477.2) [22.0 to 70000.0]	125 (17.9%)
Haemoglobin A1c (mmol/mol)	50.5 (15.6) [26.0 to 115.0]	137 (19.6%)
**Baseline medication**		
Antiplatelet use		1 (0.1%)
None	152 (21.8%)	
Single antiplatelet therapy	339 (48.5%)	

Dual antiplatelet therapy	208 (29.8%)	
Anticoagulation use	232 (33.1%)	0 (0%)
Beta-blocker use	634 (90.6%)	0 (0%)
ACE-inhibitor use	472 (67.7%)	3 (0.4%)
Angiotensin II receptor blocker use	115 (16.6%)	7 (1.0%)
Aldosterone antagonist use	346 (49.6%)	3 (0.4%)
Loop or thiazide diuretics use	460 (66.0%)	3 (0.4%)
Statin use	601 (85.9%)	0 (0%)
Amiodarone use	34 (4.9%)	1 (0.1%)
Digoxin use	52 (7.4%)	1 (0.1%)

Numbers in table is mean (standard deviation) [range], median (interquartile range) [range] or frequencies (percentage) as appropriate.

PCI – Percutaneous coronary intervention, CCS – Canadian Cardiovascular Society, NYHA – New York Heart Association, ECG – Electrocardiogram, BCIS – British Cardiovascular Intervention Society, IHD – Ischaemic heart disease, CABG – Coronary artery bypass graft, HF – Heart failure, NT-proBNP – N-terminal fragment of the prohormone brain-type natriuretic peptide, ACE – Angiotensin converting enzyme

Supplementary table 2: Univariable Cox proportional hazards models

Supplementary table 2: Univariable Cox proportional hazards models for time to all-cause death or hospitalisation due to heart failure

Predictor	HR	95% CI	*p*-value*
**Trial allocation**			0.96
Optimal medical therapy alone	1.00	Reference	
PCI plus optimal medical therapy	0.99	0.78 to 1.27	
**Baseline general characteristics**			
Age at randomisation (per 5 year)	1.12	1.05 to 1.21	<0.001
Male gender†	1.20	0.80 to 1.81	0.36
Body weight (per 5kg increase)	0.98	0.95 to 1.02	0.38
Height (per 5cm increase)	1.04	0.97 to 1.12	0.30
Body Mass Index (kg/m^2)			0.002
<25	1.00	Reference	
>25 to <30	0.58	0.43 to 0.78	
>30	0.71	0.53 to 0.95	
Ethnicity			0.29
Caucasian	1.00	Reference	
Non-Caucasian	1.25	0.84 to 1.84	
**Baseline clinical characteristics**			
Log of systolic BP (per log[mmHg] increase)	0.32	0.15 to 0.71	0.005
Log of diastolic BP (per log[mmHg] increase)	0.28	0.13 to 0.57	<0.001
Log of mean arterial BP (per log[mmHg] increase)	0.22	0.09 to 0.51	<0.001
Heart rate (per 5 beats/minute increase)	0.97	0.92 to 1.02	0.18
Canadian Cardiovascular Society Angina Grade			0.40
Grade 0	1.00	Reference	
Grade 1 or 2	0.92	0.71 to 1.20	
Grade 3 or 4	1.55	0.76 to 3.16	
New York Heart Association Classification			<0.001
Grade I or II	1.00	Reference	
Grade III or IV	2.04	1.59 to 2.62	
Left ventricular ejection fraction (per 5% increase)	0.98	0.91 to 1.05	0.56
Atrial fibrillation on baseline ECG†	1.03	0.75 to 1.42	0.86
Number of atherosclerotic lesions to be treated (per lesion increase)	1.08	0.99 to 1.18	0.10
BCIS Jeopardy score (per 2 point increase)	1.07	0.96 to 1.18	0.20
Smoking history			0.88
Never smoker	1.00	Reference	

Ex-smoker	1.01	0.76 to 1.36	
Current smoker	1.09	0.76 to 1.56	
Hypertension	1.42	1.11 to 1.82	0.005
Hypercholesterolaemia	1.22	0.95 to 1.56	0.11
Peripheral vascular disease	1.96	1.45 to 2.65	<0.001
Diabetes	1.60	1.25 to 2.05	<0.001
Dialysis treatment	5.47	2.80 to 10.72	<0.001
Family history of premature IHD	1.15	0.90 to 1.48	0.26
Previous cerebrovascular disease	1.17	0.82 to 1.66	0.40
Previous myocardial infarction	1.12	0.87 to 1.42	0.38
Previous percutaneous coronary intervention	1.09	0.81 to 1.48	0.56
Previous coronary artery bypass graft	1.30	0.77 to 2.19	0.34
Admission due to HF (previous 2 years)	1.63	1.27 to 2.08	<0.001
**Baseline biochemistry**			
Log of haemoglobin (per log[g/L] increase)	0.13	0.05 to 0.32	<0.001
Log of creatinine (per log[μmol/L] increase)	3.24	2.38 to 4.40	<0.001
Total cholesterol (per mmol/L increase)	0.87	0.78 to 0.99	0.025
Low-density lipoprotein (per mmol/L increase)	0.83	0.69 to 0.99	0.035
High-density lipoprotein (per mmol/L increase)	0.86	0.58 to 1.28	0.47
Triglyceride (per mmol/L increase)	0.88	0.77 to 1.01	0.063
Log of NT-proBNP (per log[ng/L] increase)	1.63	1.45 to 1.82	<0.001
Haemoglobin A1c (per 5 mmol/mol increase)	1.07	1.03 to 1.12	<0.001
**Baseline medication†**			
Antiplatelet use			0.29
No use	1.00	Reference	
Single antiplatelet therapy	1.07	0.77 to 1.47	
Dual antiplatelet therapy	1.28	0.91 to 1.80	
Anticoagulant use	1.07	0.83 to 1.39	0.59
Beta-blocker use	0.75	0.51 to 1.11	0.17
ACE-inhibitor use	1.03	0.79 to 1.34	0.81
Angiotensin II receptor blocker use	0.85	0.61 to 1.19	0.34
Aldosterone antagonist use	0.79	0.62 to 1.01	0.057
Loop or thiazide diuretics use	1.65	1.25 to 2.18	<0.001
Statin use	1.35	0.92 to 2.00	0.11
Amiodarone use	1.14	0.68 to 1.92	0.63
Digoxin use	0.89	0.55 to 1.44	0.64

HR – Hazard ratio, CI – Confidence interval, PCI – Percutaneous coronary intervention, BP – Blood pressure, BCIS – British Cardiovascular Intervention Society, ECG – Electrocardiogram, IHD –

Ischaemic heart disease, HF – Heart failure, NT-proBNP – N-terminal fragment of the prohormone brain-type natriuretic peptide, log – Natural log-transformation.

* *p*-values based on likelihood ratio test.

† If the reference level is not directly indicated the absence of the factor will be reference level.

Supplementary table 3: Performance statistics of the stepwise prediction model

Supplementary table 3: Performance of the stepwise prediction model

	Est (95% CI)	Oc-est (95% CI)
Harrell's C – 2 y	0.74 (0.70 to 0.78)	0.70 (0.66 to 0.74)
Harrell's C – 4 y	0.73 (0.70 to 0.76)	0.69 (0.66 to 0.73)
Harrell's C – full follow-up	0.72 (0.69 to 0.76)	0.69 (0.66 to 0.72)
Gönen and Hellers K – 2 y	0.69 (0.67 to 0.72)	0.69 (0.67 to 0.72)
Gönen and Hellers K – 4 y	0.69 (0.67 to 0.72)	0.69 (0.67 to 0.72)
Gönen and Hellers K – full follow-up	0.69 (0.67 to 0.72)	0.70 (0.67 to 0.72)
Scaled Brier score – 2 y	0.16 (0.09 to 0.23)	0.09 (0.03 to 0.16)
Scaled Brier score – 4 y	0.19 (0.11 to 0.26)	0.10 (0.03 to 0.18)
Scaled Brier score – 6 y	0.18 (0.07 to 0.29)	0.10 (-0.02 to 0.21)
Calibration slope	1.00 (0.85 to 1.15)	0.79 (0.64 to 0.95)

Est – Apparent performance estimate, Oc-est – Optimism-corrected estimate, y – years of follow-up

All estimates calculated in each imputed dataset and combined using Rubin's rules.

Supplementary table 4: Effect of trial intervention stratified by baseline risk of primary outcome (5 cut points – stepwise prediction model)

Supplementary table 4: Effect of trial intervention stratified by baseline risk of primary outcome (5 cut points)

Risk groups	Cut points		HR (95% CI)*	*p* for interaction
	Risk score	Predicted 6-year event probability		
				0.29
Lowest risk	<5.97	<39.8%	0.73 (0.40 to 1.35)	
Low medium risk	5.97 to 6.44	39.8% to 55.5%	0.84 (0.44 to 1.59)	
Medium risk	6.44 to 6.86	55.5% to 71.2%	0.93 (0.49 to 1.76)	
High medium risk	6.86 to 7.27	71.2% to 84.5%	1.80 (0.98 to 3.30)	
Highest risk	>7.27	>84.5%	1.25 (0.70 to 2.24)	

No.— Number, CI— Confidence intervals, HR – hazard ratio

Data in table are based on 10 imputed datasets. Risk groups are formed to allow approximate equal number of events in each group.

* Hazard ratios <1.00 favour PCI plus OMT.

Supplementary table 5: Final LASSO prediction model for the primary outcome

Supplementary table 5: Final LASSO prediction model for the primary outcome (all-cause death or hospitalisation due to heart failure) (n=700)

Predictor	β	HR
Age at randomisation (per 5 year)	0.063	1.06
BCIS Jeopardy score (per 2 point increase)	0.036	1.04
Left ventricular EF (per 5% increase)	0.035	1.04
Heart rate (per 5 beats/minute increase)	-0.019	0.98
Peripheral vascular disease	0.385	1.47
New York Heart Association Classification		
Grade I or II	0.000	1.00
Grade III or IV	0.282	1.33
Admission due to HF (previous 2 years)	0.208	1.23
Log of NT-proBNP (per log[ng/L] increase)	0.316	1.37
Haemoglobin A1c (per 5 mmol/mol increase)	0.039	1.04
Log of creatinine (per log[µmol/L] increase)	0.452	1.57
Loop or thiazide diuretics use	0.149	1.16
Beta-blocker use	-0.212	0.81
Male gender	0.024	1.02
Body Mass Index (kg/m^2)		
<25 or >30	0.000	1.00
>25 to <30	-0.110	0.90
Log of mean arterial BP (per log[mmHg] increase)	-0.381	0.68
Smoking history		
Never or Ex-smoker	0.000	1.00
Current smoker	0.031	1.03
Family history of premature IHD	0.007	1.01
Hypertension	0.033	1.03
Hypercholesterolaemia	0.019	1.02
Total cholesterol (per mmol/L increase)	-0.012	0.99
Angiotensin II receptor blocker use	-0.021	0.98

β - beta coefficients from the model, HR – hazard ratio, BCIS – British Cardiovascular Intervention Society, EF – ejection fraction, BP – blood pressure, IHD – ischaemic heart disease, NT-proBNP – N-terminal fragment of the prohormone brain-type natriuretic peptide, log – Natural log-transformation. Baseline survival probabilities (LASSO model): $S_0(2) = 0.9967$ (2-year), $S_0(4) = 0.9937$ (4-year) and $S_0(6) = 0.9888$ (6-year)

Supplementary table 6: Performance statistics of the LASSO prediction model

Supplementary table 6: Performance statistics of the LASSO prediction model

	Est (95% CI)	Oc-est (95% CI)
Harrell's C	0.73 (0.70 to 0.76)	0.70 (0.66 to 0.73)
Gönen and Hellers K	0.66 (0.65 to 0.67)	0.66 (0.65 to 0.67)
Scaled Brier score – 2 y	0.16 (0.11 to 0.21)	0.10 (0.05 to 0.15)
Scaled Brier score – 4 y	0.19 (0.13 to 0.24)	0.12 (0.06 to 0.17)
Scaled Brier score – 6 y	0.17 (0.08 to 0.27)	0.10 (0.00 to 0.19)
Calibration slope	1.25 (1.05 to 1.45)	1.04 (0.84 to 1.25)

Est – Apparent performance estimate (across imputed datasets), Oc-est – Optimism-corrected estimate, y – years of follow-up

All estimates calculated in each imputed dataset and combined using Rubin's rules.

Supplementary table 7: Effect of trial intervention stratified by baseline risk of primary outcome (LASSO prediction model)

Supplementary table 7: Effect of trial intervention stratified by baseline risk of primary outcome (LASSO prediction model)

Risk groups	Risk difference, percentage points (95% CI)*		HR (95% CI)†	*p* for interaction
	At 2 years	At 4 years		
				0.56
Low risk	1.4 (-4.5 to 7.4)	1.6 (-7.4 to 10.6)	0.87 (0.56 to 1.34)	
Medium risk	-5.4 (-21.1 to 10.2)	5.0 (-14.0 to 24.2)	0.97 (0.59 to 1.60)	
High risk	16.8 (-4.1 to 37.6)	5.4 (-15.5 to 26.2)	1.22 (0.79 to 1.89)	

No.–- Number, CI–- Confidence intervals, HR – hazard ratio

Data in table are based on 10 imputed datasets. Risk groups are formed to allow approximate equal number of events in each group.

*Absolute risk difference based on the Kaplan-Meier estimator (negative values favour percutaneous coronary intervention [PCI]) plus optimal medical therapy [OMT]). The 95% Confidence intervals are based on 500 bootstrap replications in each imputed dataset.

† Hazard ratios <1.00 favour PCI plus OMT.

SUPPLEMENTARY FIGURES

Supplementary figure 1: Apparent calibration for the stepwise prediction model

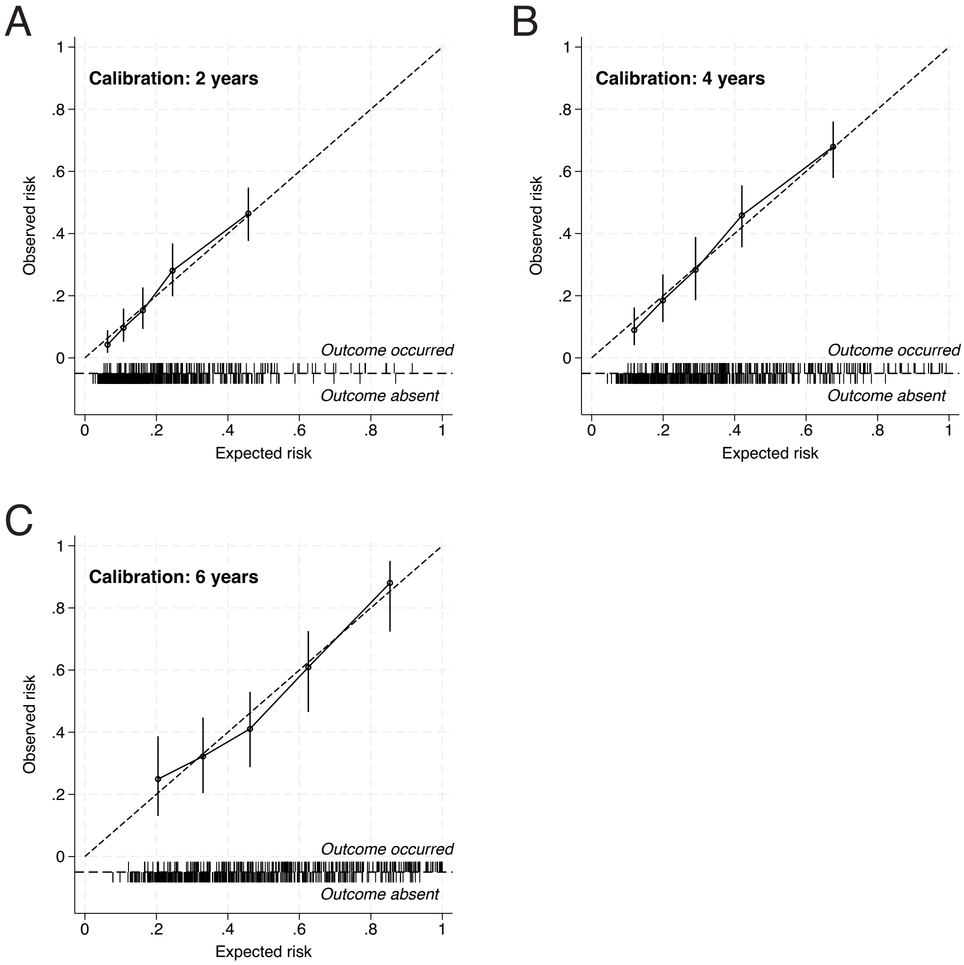

Panel A, B and C depicts the calibration plots at 2, 4 and 6 years after randomisation (combined over imputed datasets). In each calibration plot, the calibration for five groups of participants is displayed with the average predicted risk plotted against the observed (95% confidence intervals). The spike-plots presented at the bottom of the calibration plots indicates, for each participant, the mean predicted risk across the imputed datasets at 2, 4 and 6 years respectively along with their individual outcome status.

Supplementary figure 2: Distribution of baseline risk score and 6-year event probability as predicted by the stepwise prediction model

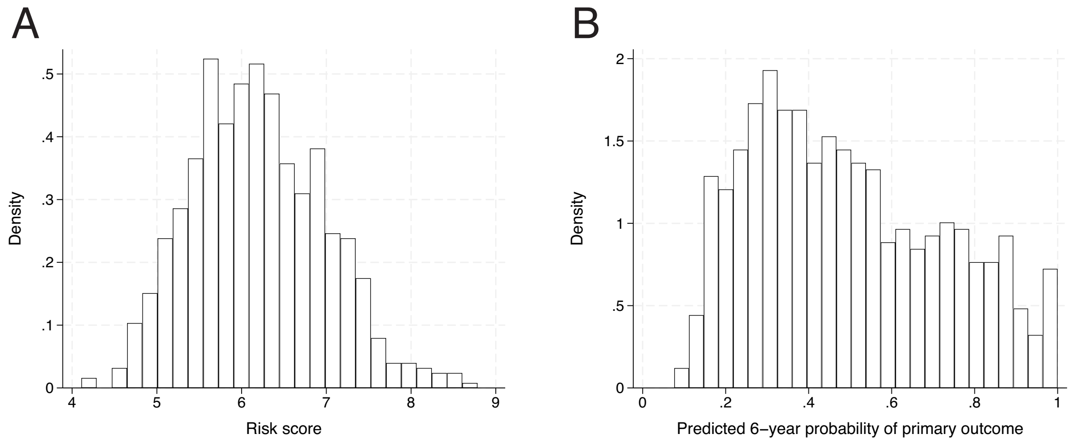

Panel A depicts the distribution of the predicted risk score across the REVIVED-BCIS2 trial population. Panel B depicts the distribution of the predicted 6-year probability of suffering all-cause mortality or hospitalisation due to heart failure across the REVIVED-BCIS2 trial population. The participants risk scores and predicted 6-year event probabilities were combined across imputed datasets using Rubin's rule.

Supplementary figure 3: Cumulative probability of primary outcome by allocation and risk groups as predicted by the stepwise prediction model

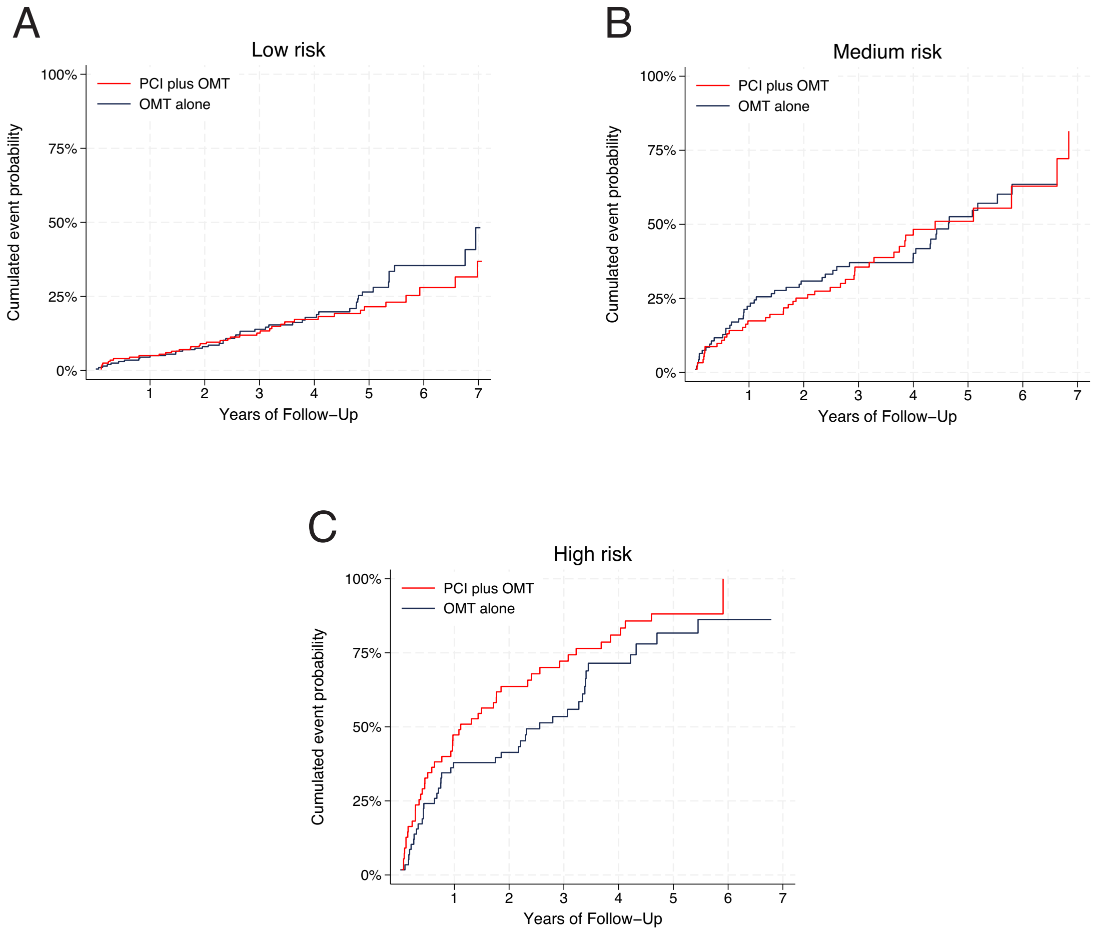

Cumulative probability of all-cause mortality or hospitalisation due to heart failure among participants allocated to percutaneous coronary intervention (PCI) plus optimal medical therapy (OMT) or OMT alone across the follow-up. Participants are separated into three risk strata based on their predicted baseline risk of the all-cause mortality or hospitalisation due to heart failure – a low risk group (panel A), a medium risk group (panel B) and a high risk group (panel C).

Supplementary figure 4: Apparent calibration for the LASSO prediction model

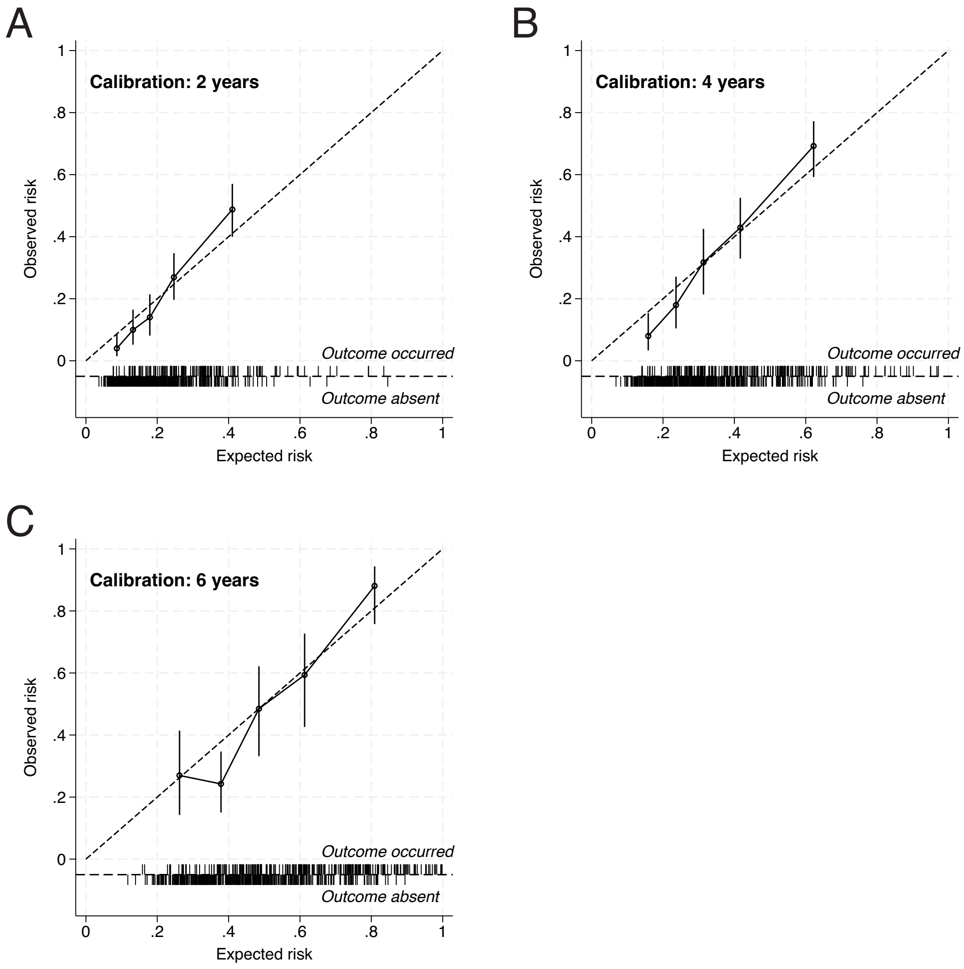

Panel A, B and C depicts the calibration plots at 2, 4 and 6 years after randomisation (combined over imputed datasets). In each calibration plot, the calibration for five groups of participants is displayed with the average predicted risk plotted against the observed (95% confidence intervals). The spike-plots presented at the bottom of the calibration plots indicates, for each participant, the mean predicted risk across the imputed datasets at 2, 4 and 6 years respectively along with their individual outcome status.

Supplementary figure 5: Intervention effect for different levels of baseline risk predicted by LASSO model

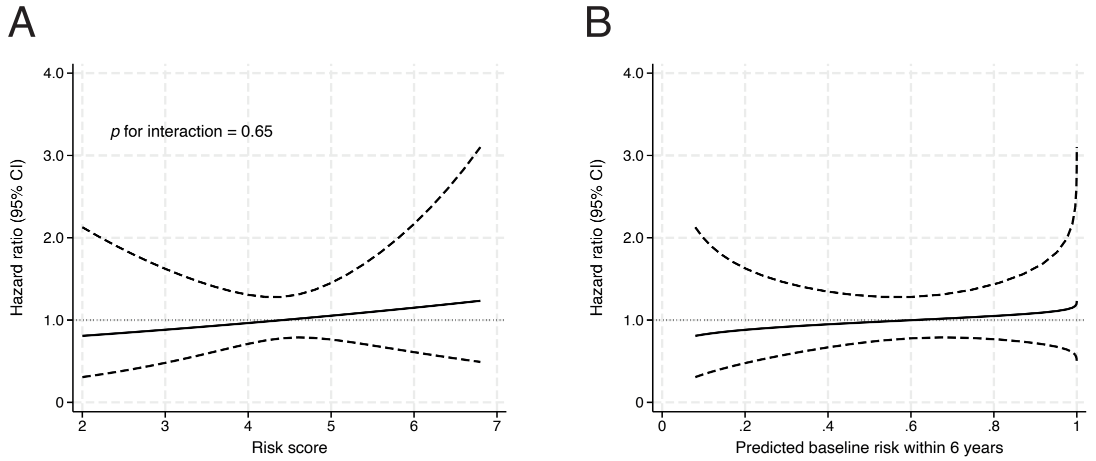

The intervention effect (solid line) and 95% confidence intervals (dashed lines) of percutaneous coronary intervention plus optimal medical therapy (OMT) versus OMT alone for different levels of the risk score (panel A) or the predicted baseline risk of the primary outcome after 6 years of follow-up (panel B). A hazard ratio <1.00 favour PCI plus OMT.

Supplementary figure 6: Cumulative probability of primary outcome by allocation and risk groups as predicted by the LASSO model

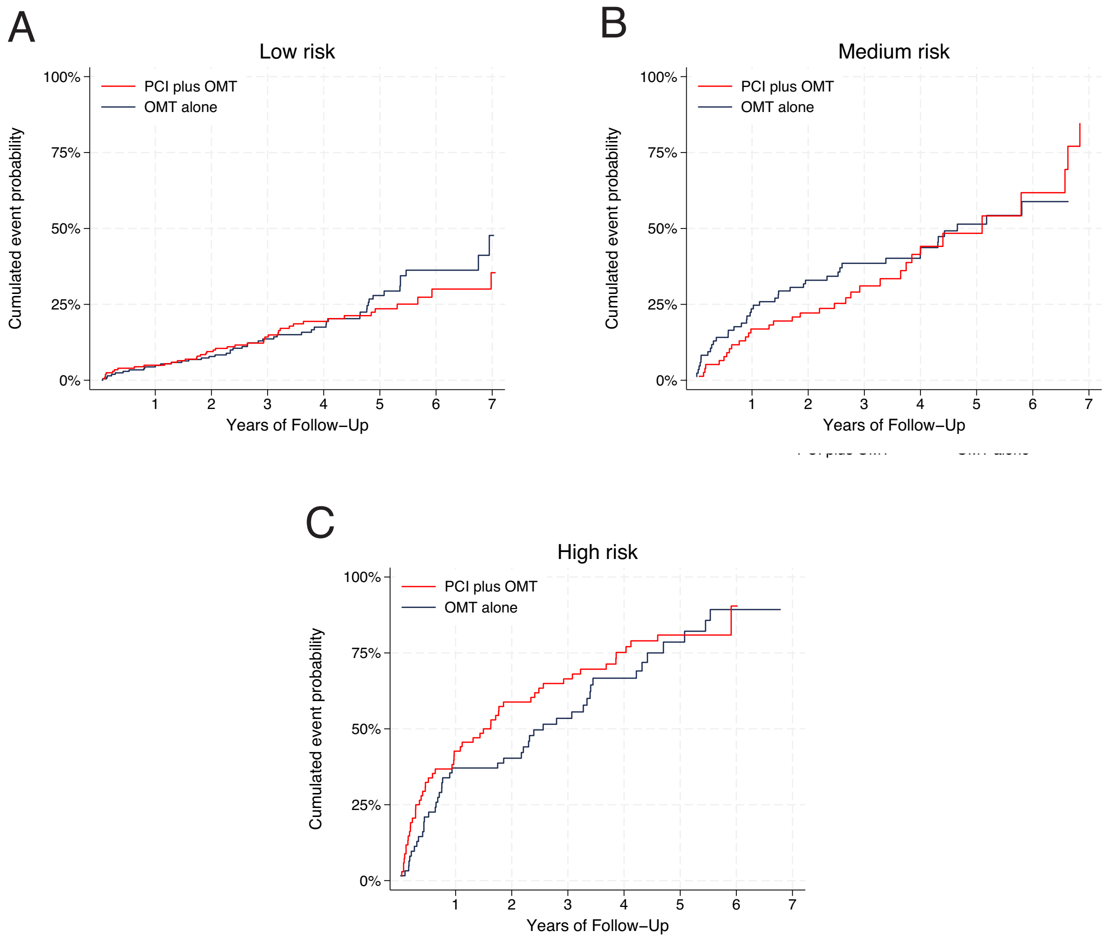

Cumulative probability of all-cause mortality or hospitalisation due to heart failure among participants allocated to percutaneous coronary intervention (PCI) plus optimal medical therapy (OMT) or OMT alone across the follow-up. Participants are separated into three risk strata based on their predicted baseline risk of the all-cause mortality or hospitalisation due to heart failure – a low risk group (panel A), a medium risk group (panel B) and a high risk group (panel C).

SUPPLEMENTARY REFERENCES

1. Moons KG, Donders RA, Stijnen T, Harrell FE, Jr. Using the outcome for imputation of missing predictor values was preferred. *J Clin Epidemiol*. 2006;59:1092-1101.
2. White IR, Royston P. Imputing missing covariate values for the Cox model. *Stat Med*. 2009;28:1982-1998.
3. Wood AM, White IR, Royston P. How should variable selection be performed with multiply imputed data? *Stat Med*. 2008;27:3227-3246.
4. Greenland S. Modeling and variable selection in epidemiologic analysis. *Am J Public Health*. 1989;79:340-349.
5. Steyerberg EW. *Clinical prediction models : a practical approach to development, validation, and updating*. Second ed. Switzerland: Springer; 2019.
6. Harrell FE, Jr., Lee KL, Mark DB. Multivariable prognostic models: issues in developing models, evaluating assumptions and adequacy, and measuring and reducing errors. *Stat Med*. 1996;15:361-387.
